# 105 Nursing Theory and Burn Competency Training Practices to Address Gaps in Post-covid Trained Graduate Nurses

**DOI:** 10.1093/jbcr/irac012.108

**Published:** 2022-03-23

**Authors:** Lynn Dowling, Joan Wilson, Jane D Echols, Susan Burke, Jocelyn Hills

**Affiliations:** Joseph M. Still Burn Center at Doctors Hospital, Augusta, Georgia; Joseph M. Still Burn Center at Doctors Hospital, Augusta, Georgia; Joseph M. Still Burn Center at Doctors Hospital, Augusta, Georgia; Doctors Hospital Augusta, Augusta, Georgia; Burn & Reconstructive Centers of America, Augusta, Georgia

## Abstract

**Introduction:**

Recruitment efforts just after the recent COVID crises brought in several new graduate nurses. They had limited clinical exposure during COVID-19 resulting in difficulty transitioning into practice providing safe patient care. As a result, these nurses lacked the fundamental knowledge needed to care for acutely ill burn and wound patients resulting in the new graduate registered nurses (NGRNs) feeling overwhelmed at the bedside. These findings coincide with assessments noted in Kavanagh and Sharpnack’s (2021), article identifying only 9% of NGRNs were practice ready, with 7% failing to recognize urgency or a change in a patient’s condition.

**Methods:**

In order to achieve the designated American Burn Association (ABA) competencies, our center designed a program based on Patricia Benner’s Novice to Expert nursing theory. Additionally, we divided the competencies into achievable goals and domains using the Donna Wright's nursing competency model.

StaRN program: didactics/simulation/skills/unit orientation one on one with a preceptor

Competency based staged orientation program for new staff

Burn Specific Education includes:

1) Burn and complex wound care didactic

2) Burn specific High-fidelity simulation scenario utilizing critical care equipment promoting critical thinking and critical reasoning skills

3) Task trainers

4) On-going preceptor education

5) Nurse Extern program

**Results:**

NGRNS arriving at our unit in early 2020 were found to be incapable of performing clinical tasks in the burn ICU (BICU) setting at the level of competency recommended by the ABA. We immediately placed this cohort into the revised training program incorporating Benners Novice to Expert Theory and Wright’s Competency Model. Of the 25, 17 were able to be placed in the BICU (68%), and 8 were able to transfer to a lower level of care (progressive care/med-surg). All 25 were given extended orientation (12 weeks instead of the normal 8, as recommended by our facility). We will follow this group to determine retention rates.

**Conclusions:**

Current levels of competencies by the ABA creates gaps in care for graduate nurses entering the workforce with deficits. Applying Benner's Novice to Expert Theory and Wright’s Competency Model to modify approaches to training helps identify gaps in care, addresses areas that are weak for the nurse, and help guide the graduate nurse through stages of expertise to arrive more confidently at the level of competency expected by the ABA.

